# Moisture variation inferred from tree rings in north central China and its links with the remote oceans

**DOI:** 10.1038/s41598-021-93841-1

**Published:** 2021-08-12

**Authors:** Zeyu Zheng, Liya Jin, Jinjian Li, Jie Chen, Xiaojian Zhang, Zhenqian Wang

**Affiliations:** 1 MOE Key Laboratory of Western China’s Environmental System, College of Earth and Environmental Sciences, Lanzhou University, Lanzhou, 730000 Gansu China; 2 School of Atmospheric Sciences, Chengdu University of Information Technology, Chengdu, 610225 Sichuan China; 3 School of Geographic and Oceanographic Sciences, Nanjing University, Nanjing, 210023 Jiangsu China

**Keywords:** Climate change, Palaeoclimate

## Abstract

In this study we presented a composite standard chronology, spanning 1635–2018 to reconstruct May–July moisture variation in north central China. Our reconstruction revealed four severe dry epochs and five pronounced wet epochs. Additionally, spatial correlation analysis of our reconstruction with the actual self-calibrating Palmer drought severity index showed that our reconstruction was representative of large-scale May–July moisture changes. Both the severe dry and pronounced wet epochs showed one-to-one correspondence with other reconstructions nearby during their common periods, which demonstrated the reliability of our reconstruction backwards in time. Spectral analysis showed that significant spectral peaks were found at 2.1–3.8 years, which fell within the overall bandwidth of the El Niño-Southern Oscillations (ENSO). The spatial correlation patterns between our reconstruction and sea surface temperature (SST) in the equatorial eastern Pacific further confirmed the link between regional moisture and ENSO, with warm-phase ENSO resulting in low moisture and vice-versa. However, this link was time-dependent during the past four centuries, and was modulated by different phases of SST in the tropical Indian Ocean. Additionally, significant peaks at 24.9–46.5 years and spatial correlation patterns indicated that the Pacific Decadal Oscillation and the North Atlantic Oscillation may be the possible forcing factors of regional moisture at lower frequencies.

## Introduction

In recent decades, drought occurrence has been likely to increase in intensity and frequency under the background of global climate change^1–4^. This kind of extreme event has a significant impact on ecosystems, the social economy and the local populations because of its close relationship with water sources, agricultural production and so on^5,6^. For example, severe drought occurred during the 1920s in northern China, in the 1930s on the Great Plains of the United States, in the 1980s in Africa’s Sahel region and in recent decade in the region the Americas, resulting in mass deaths and economic losses^7–11^. These severe drought occurrences highlight the importance of understanding the relative roles of hydroclimate variations and their forcing mechanisms^12^. However, the observed records are limited in time and space. In China, most meteorological stations were established in the east after the 1960s^13,14^, which prevents us from probing into the climate change on longer time scales. Therefore, many researchers use different natural archives, such as lake sediments^15,16^, stalagmites^17,18^ and tree rings^19–21^, to decode paleoclimate change at various timescales, and they have made great progress with these methods. Tree rings, as a widespread natural archive, are extensively used because of their high resolution.

During the past decades, many dendroclimatologists have focused on north central China, a semi-humid region, and one of the most sensitive regions for climate change. In this region, fluctuations in the East Asian summer monsoon (EASM) will lead to substantial variability in regional moisture for its location near the north fringe of EASM^22^, particularly in the western part of this region, where the western Qinling Mountains stands, an important geographic demarcation line for climate^23^ and the Loess Plateau adjoins^24^. Previous studies have reconstructed the summer and annual Palmer drought severity index (PDSI)^25–29^, summer and annual precipitation^30,31^ and summer relative humidity^32,33^ during recent centuries. Most of these studies found the possible forcing mechanisms of large-scale ocean-atmospheric circulations on regional hydroclimate change^26–29,32,33^. For instance, Fang et al.^27,28^ reconstructed the May–August and annual PDSI in the Xinglong Mountain and Guiqing Mountain areas, respectively, and suggested the El Niño-Southern Oscillations (ENSO) impact on regional moisture change. Additionally, Liu et al.^32^ reconstructed the July–August relative humidity in Shimen Mountain and found ENSO is the affecting factor for the regional relative humidity variations. Wang et al.^33^ also regarded ENSO as a possible factor for summer relative humidity variations inferred from tree-ring δ^18^O originating from Gansu Province. These achievements have revealed past climate changes and identified climate anomaly during the Little Ice Age around the seventeenth century and global warming in the late twentieth century. More importantly, they have promoted our understanding of the influences of large-scale ocean-atmospheric circulations on climate change and helped us to evaluate ongoing and future climate changes in this environmentally sensitive region. However, uncertainties remain regarding whether the influence of ENSO on regional moisture variability is stable and the possible modulation factor for ENSO-hydroclimate linkage in north central China. On the one hand, previous investigators mostly focused on the influence of ENSO on regional moisture change, i.e., the moisture variability under different phases of ENSO^27,28,34^. However, because ENSO itself is a complex physical process and is loosely associated with precipitation-generating mechanisms^35^, it is quite difficult to draw a specific conclusion. An evident example is that before the late 1970s, the relationship between ENSO and summer precipitation in China was close, with the El Niño phase in previous winters corresponding to more summer rainfall in North China and south of the Yangtze River valley and vice versa. Nevertheless, weak relation between ENSO and summer precipitation was observed in China after the 1980s^36^. Therefore, it is important to detect the stability of the influence of ENSO on regional moisture variability over centuries. On the other hand, ENSO is widely used as an important predictor for severe flood and drought events^36^. Thus, investigating the possible modulation factor for ENSO-hydroclimate linkage in the past would decrease the difficulty of regional climate prediction in north central China.

Here, we presented a composite chronology, originating from Guiqing Mountain (GQM) and Shimen Mountain (SMM) in north central China to address the gap with aims of: (1) revealing the regional moisture variation during the past four centuries; (2) detecting the influence of ENSO on regional moisture variability; and (3) investigating linkages between regional moisture with ENSO and the possible modulation factor for the ENSO-hydroclimate in north central China. In the context of global climate change, this study provides a reference for regional moisture variation in past centuries in north central China and interprets the relevant possible forcing mechanisms. In particular, this study investigated the possible modulation factor for the ENSO-hydroclimate linkage, which is beneficial for regional climate prediction.

## Results

### Climate-growth response

**Figure 1:**
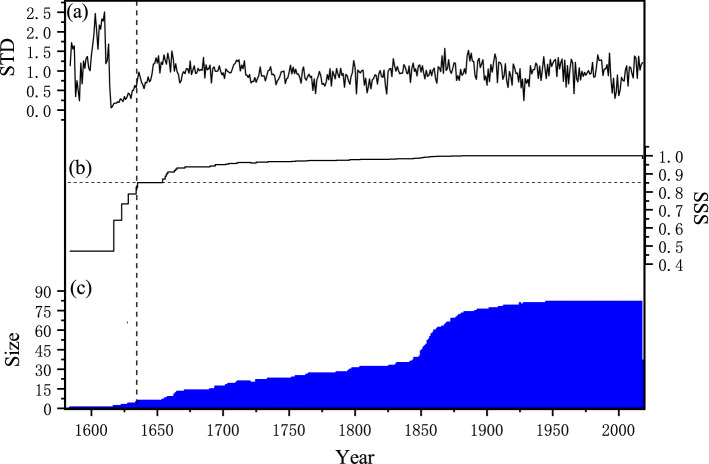
(**a**) The composite chronology developed from GQM and SMM; (**b**) the running SSS statistics. The horizontal dashed line denotes the 0.85 cutoff value; (**c**) the corresponding sample size. The vertical dashed line denotes when SSS > 0.85, the corresponding reliable STD and the sample size.

A 434-year (1584–2017) and a 396-year (1623–2018) chronology from the GQM and SMM sites were developed, respectively. Subsample signal strength (SSS)^37^ higher than 0.85 was selected to determine the reliable reconstruction period. These two chronologies agreed very well with each other, with a correlation coefficient of 0.45 (n = 360, *p* < 0.0001) at the common reliable period of 1658–2017. Further considering their close location and high environmental homogeneity, we gathered all ring width indices and used the program ARSTAN to generate a composite regional standard chronology (STD). This composite STD (hereafter GS) was reliable for the period of 1635–2018 when the sample size exceeded 6 cores (Fig. 1). The relevant statistical characteristics of the standard tree-ring chronology are shown in Table 1.

**Table 1:** Statistical characteristics of GS chronology.

Statistical item	Chronology
Standard deviation (SD)	0.326
Mean sensitivity (MS)	0.235
First order autocorrelation (AR_1_)	0.623
Common interval	1900–2018
%Variance of 1st PC(PC1)	47.9%
Signal to noise ratio (S/N)	64.8
Expressed population signal (EPS)	0.984
First year of SSS > 0.85 (number of cores)	1635 (6)

**Figure 2:**
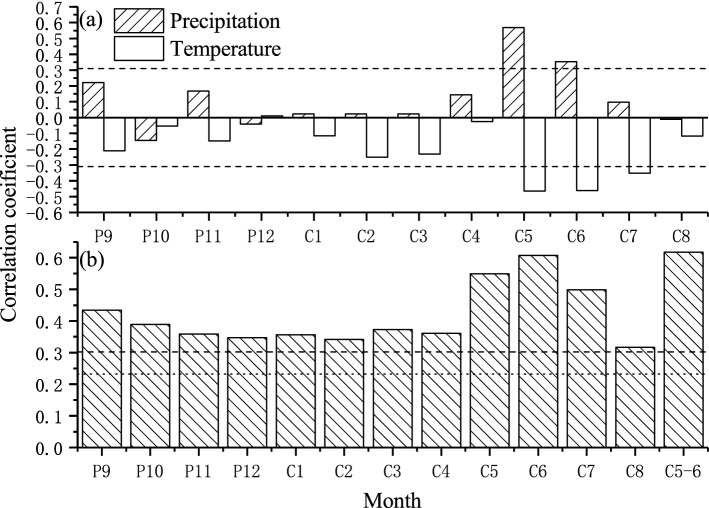
Correlations of GS with (**a**) monthly total precipitation and monthly mean temperature data for 1951–2018; (**b**) scPDSI for 1943–2014. The dashed lines indicate the corresponding 99% confidence levels.

Correlation analysis was conducted to probe the relationship of tree growth with climatic factors. Because tree growth is affected by the conditions from the previous year to the current growing season, the Pearson correlation coefficients were calculated from the previous September to the current August for 1951–2018. The correlation coefficients of GS with climate factors are shown in Fig. 2a, and statistically significant (p < 0.01) positive correlations with precipitation were observed from May to June. Significant negative correlations with temperature were found from current May to July. These results indicate a typical moisture stress on tree growth^27,28,34,38,39^. We therefore examined the correlation of GS with the self-calibrating Palmer drought severity index (scPDSI) during their common period for 1943–2014. As shown in Fig. 2b, statistically significant (p < 0.01) positive correlations with the scPDSI were found in all months from the previous September to the current August, with the highest value (0.61) observed in June. Additionally, the correlation coefficients in May and July were high, with values of 0.55 and 0.5, respectively. Because seasonal climate condition is vital for tree growth, we calculated the correlation coefficients among GS and different month combinations and found that the highest value (0.62) was in May–July. This result suggested that moisture variation in the warm season (May–July) is the most critical for Chinese pine growth in north central China.

**Figure 3:**
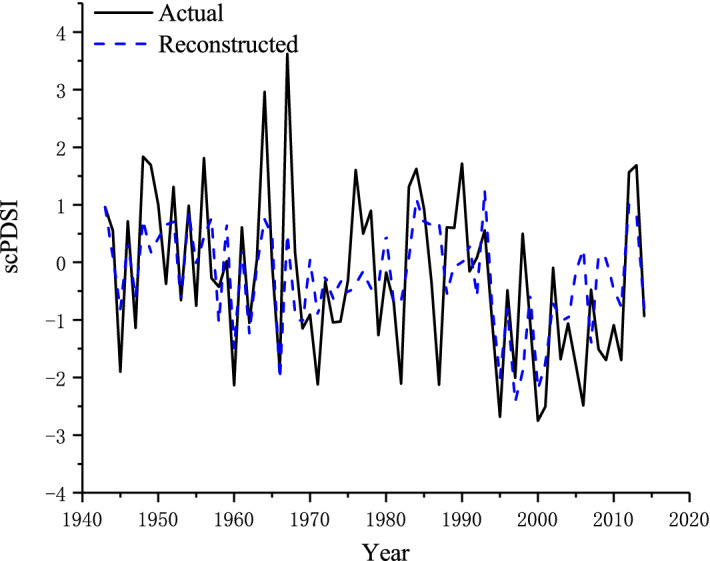
Comparison of reconstructed scPDSI with actual scPDSI for 1943–2014.

Then we built the reconstruction of the May–July scPDSI using a simple linear regression model: scPDSI_5–7_ = 3.1745 × GS − 3.3709, where scPDSI_5-7_ represents the scPDSI from May to July. The reconstruction accounted for 38.2% (R^2^adj = 0.378, *F* = 43.18) of the scPDSI variation. Then we assessed its fidelity using the split calibration-verification tests method^40^. As shown in Table 2, at the early calibration and verification period (1943–1978), the simple correlation coefficient (R), the sign test of the first-order difference (ST1) and the t-stat were 0.69, 29 and 4.09, respectively, all over a 99% confidence level, which indicated a good model fit on a high- frequency scale but less accuracy on a low-frequency scale during this period. Additionally, the calibration and verification results of the late period (1979–2014) showed better mode accuracy on the low-frequency scale but less accuracy on the high- frequency scale. Nevertheless, the calibration and verification results of the full period were ideal. The R, s sign test (ST), ST1 and t-stat were 0.62, 54, 49, and 3.42, respectively, exceeding the 99% confidence level, which manifested good model skill for the reconstruction for the period of 1943–2014 at both high- and low-frequency scales^41^. In addition, the visual comparison shown in Fig. 3 shows that the reconstruction tracks the actual scPDSI values well during the period of 1943–2014. Based on the test and visual comparison results, we reconstructed the May–July moisture change for the past 384 years.

**Table 2:** Statistics of split calibration-verification test results.

Calibration			Verification						
Period	R^2^	F	Period	R	ST	ST1	RE	CE	t-stat
1943–2014	0.382	43.18	–	0.62**	54**	49**	0.38	–	3.42**
1943–1978	0.478	31.1	1979–2014	0.55**	25*	21	0.51	0.08	2.14*
1979–2014	0.297	14.4	1943–1978	0.69**	23	29**	0.71	0.25	4.09**

**p < 0.01, *p < 0.05.

### The characteristics of the reconstructed scPDSI

**Figure 4:**
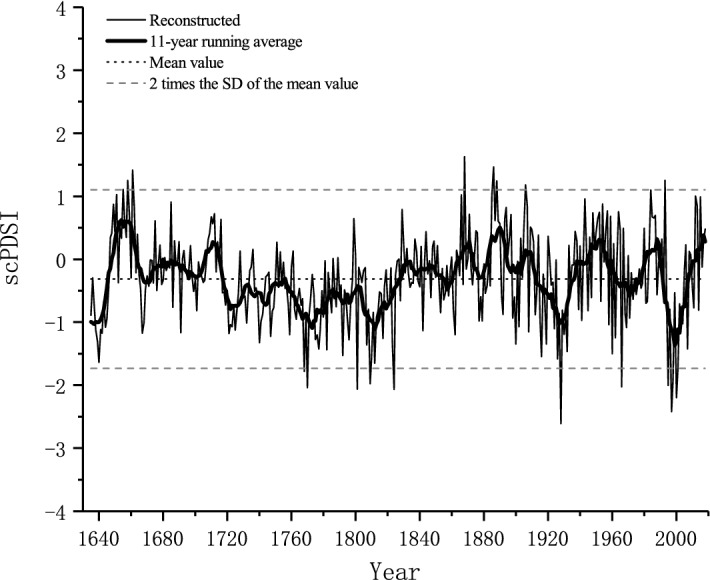
Reconstructed and 11-year running average scPDSI series.

The reconstructed scPDSI was reliable for the period of 1635–2018 and capable of resolving moisture variations on both high- and low-frequency scales. Therefore, we focused our discussion on interannual and interdecadal moisture changes (Fig. 4). According to the reconstructed scPDSI series, the mean scPDSI value was − 0.31, and the standard deviation (SD) was 0.71. In this study, we regarded the scPDSI values of − 0.31 as the normal PDSI status, and years with reconstructed values lower or greater than 2 times the SD of the mean value were considered to be extremely dry or wet conditions, respectively. Over the past 384 years, severely dry years were found in 1768, 1770, 1801, 1809, 1824, 1928, 1966, 1995, 1997, 1998, 2000, and 2001, in which 1928may have been the driest year. Extremely wet years were observed in 1655, 1658, 1661, 1868, 1886, 1888, 1903 and 1993. In general, pronounced wet conditions mainly occurred in the period prior to 1900, and the dry status seemed to occur frequently in recent decades. In addition, our reconstruction revealed marked interdecadal variation. Here we used 11-running average to detect its persistent dry/wet epochs. As shown in Fig. 4, persistent severe dry epochs occurred during the 1720–1740s, 1760–1820s, 1910–1930s and 1990–2000s, and persistent pronounced wet epochs were found in the 1650–1660s, 1670–1710s, 1830–1840s, 1860–1900s, and 1940–1950s.

**Figure 5:**
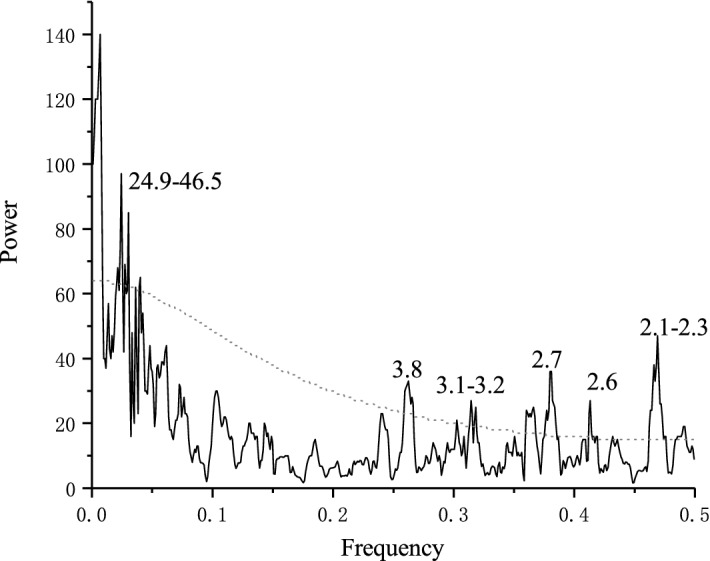
Multitaper method analysis of reconstructed scPDSI series. Dotted line denotes a 95% significant level.

The spectral power of the reconstructed scPDSI was then examined by the multi-taper method (MTM)^42^. As shown in Fig. 5, significant peaks (p < 0.05) were found at 2.1–3.8 years, which fall within the overall bandwidth of ENSO^43^. In addition, significant peaks found at 24.9–46.5 years indicated possible linkages at lower frequencies of regional moisture with the Pacific Decadal Oscillation (PDO)^44,45^ and the North Atlantic Oscillation (NAO)^46^. The details will be discussed in the following section.

## Discussion and conclusions

### Climate signal inferred from GS

The results of the climate-tree growth relationship confirmed that the growth of Chinese pine (*Pinus tabulaeformis*) in north central China is mainly controlled by the warm season moisture conditions. This type of climate-tree growth relationship is commonly found in other neighboring sites in semi-humid regions of north central China^38,47,48^.

As mentioned above, significantly positive correlations of GS with monthly total precipitation were observed only from concurrent May to June. Usually, Chinese pine is distributed in the areas where the amount of annual total precipitation is greater than 500 mm^49^. The sum of annual total precipitation (515.1 mm recorded at the Tianshui meteorological station for 1951–2019) meets the required amount for Chinese pine growth, resulting in insignificant correlations between GS and precipitation in most months. Additionally, physiological studies have revealed that more precipitation in the early growing season would trigger of xylogenesis while the temperature conditions are suitable^50^, resulting in significantly positive correlations between GS and precipitation from May to June. However, significantly negative correlations between the ring-width index and temperature were observed from the current May to July, which indicated that high temperatures led to narrow stem radius expansion. This result is because high temperatures would enhance water stress by evaporation and evapotranspiration and result in low soil moisture. When moisture drops below the threshold suitable for tree growth, the stoma will close, and therefore, low photosynthetic efficiency causes low radial growth^51,52^.

Overall, temperature and precipitation variability during the warm season influence tree growth in north central China. The scPDSI is a criterion of accumulated moisture deficit to local mean moisture conditions, which considers precipitation, temperature, ect.^53^. Therefore, using tree ring data in GS to reconstruct the May–June scPDSI is reliable.

### Spatiotemporal representativeness of the reconstructed scPDSI

**Figure 6:**
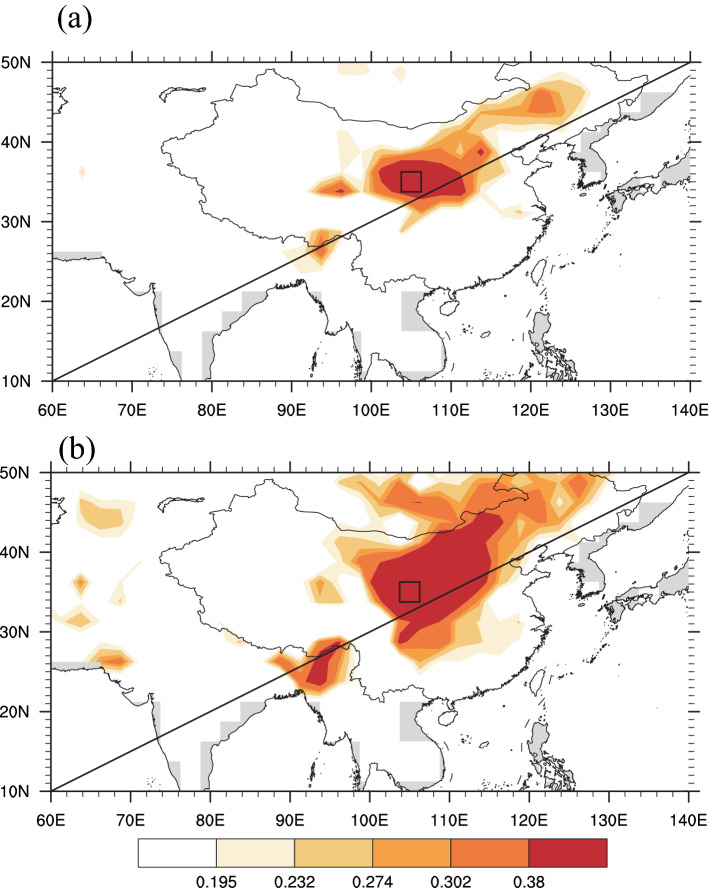
Spatial correlation patterns of (**a**) reconstructed scPDSI with actual gridded scPDSI data; (**b**) actual scPDSI with actual gridded scPDSI for 1943–2014. The black rectangle denotes our study site. Coefficients of 0.195, 0.232, 0.274, 0.302 and 0.38 denote the corresponding 90%, 95%, 98%, 99% and 99.9% confidence levels, respectively. Figures are created using NCL version 6.6.2.

To further detect the spatiotemporal representativeness of our reconstructed scPDSI, we first utilized spatial correlation analysis. As shown in Fig. 6a, the reconstructed scPDSI was significantly positive with the actual scPDSI in the surrounding area during 1943–2014, consistent with the spatial correlation pattern of the actual scPDSI correlation (Fig. 6b), which indicated that our reconstruction was representative of large-scale warm season moisture changes in north central China for the period of 1943–2014. Interestingly, these two patterns showed that reconstructed and actual scPDSI were significantly positively correlated with the scPDSI in Myanmar, where moisture change is controlled by different climate types. It may be worth further investigating possible reasons for their relationship.

**Figure 7:**
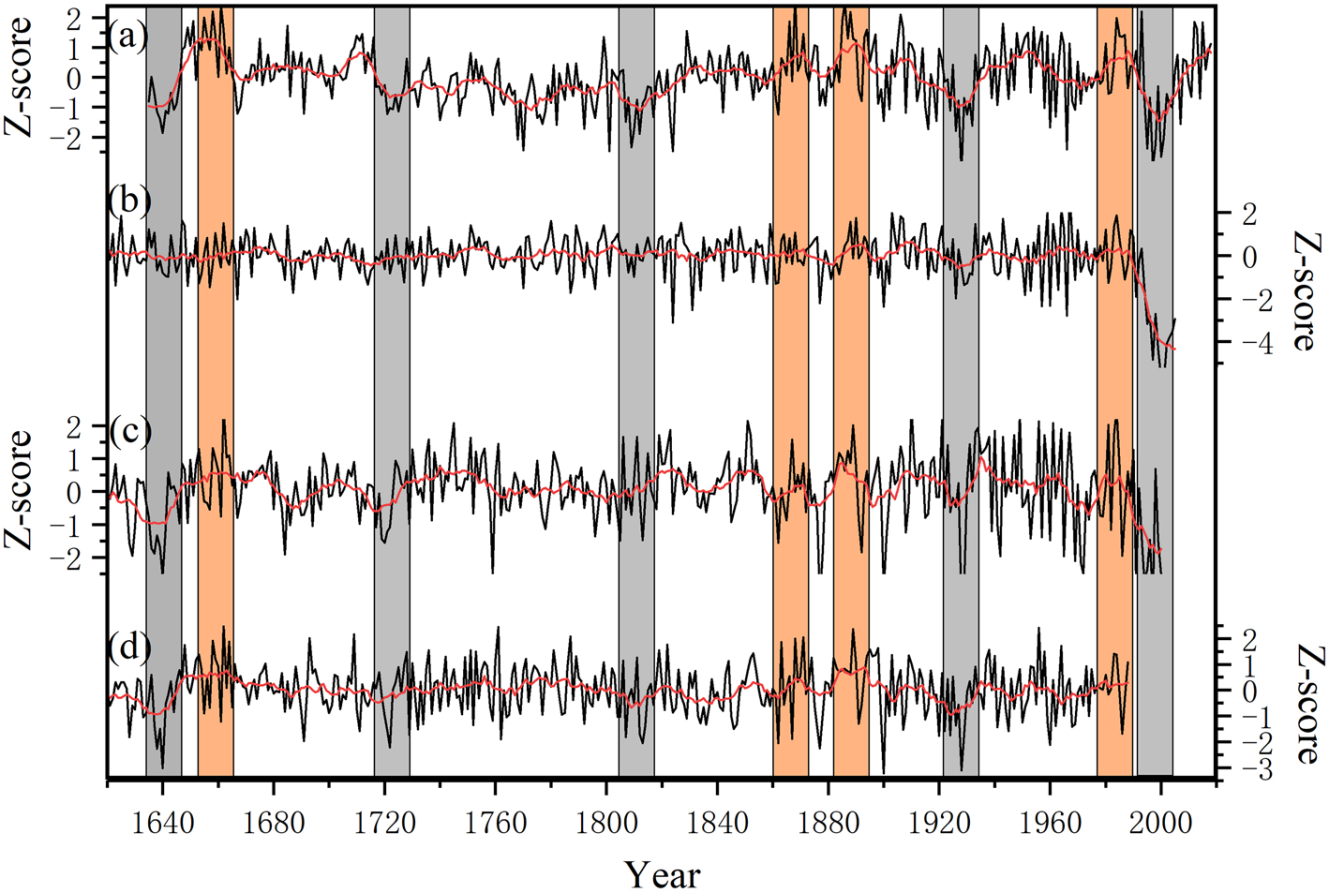
Comparison of reconstructed series with other hydroclimatic reconstructions. (**a**) Our reconstruction; (**b**) June–August PDSI series from MADA; (**c**) May–September precipitation reconstruction in the western Qinling Mountains; (**d**) April-July precipitation reconstruction in north central China. Gray and orange shades denote common severe dry and wet epochs, respectively. This is created using Origin 2021 for trial version.

Then, we compared our reconstruction with other hydroclimatic reconstructions based on tree-ring data nearby to detect the reliability of our reconstruction backwards in time. On the interannual timescale, our reconstruction (Fig. 7a) was significantly correlated with the May–July PDSI from the Monsoon Asia Drought Atlas (MADA; r = 0.45; p < 0.01; 1635–2005; Fig. 7b), May–September precipitation reconstruction in the western Qinling Mountains^31^ (r = 0.36; p < 0.01; 1635–2000; Fig. 7c) and April-July precipitation reconstruction in north central China^30^ (r = 0.41; p < 0.01; 1635–1988; Fig. 7d). On the interdecadal timescale, our record (Fig. 7a) agreed well with these three reconstructions, with significant correlation coefficients (p < 0.01) of 0.34, 0.4 and 0.51 with May–July PDSI (Fig. 7b)_,_ May–September precipitation reconstruction in the western Qinling Mountains (Fig. 7c) and April-July precipitation reconstruction in north central China (Fig. 7d), respectively. In addition, the severe dry and pronounced wet epochs recorded in our reconstruction showed one-to-one correspondence with these reconstructions. Notably, the amplitude of May–July PDSI was smaller than that in the other three reconstructions, which might be attributed to the applicability of the PDSI in China. The PDSI was developed in the central and western Great Plains, and the applicability of the PDSI in different regions is still unclear due to differences in climate types and moisture deficits.

Furthermore, our reconstruction revealed several severe dry and pronounced wet epochs that were also found in other nearby moisture records. For example, the pronounced pluvial in the 1890s and 1980s were in accordance with that in the Qilian Mountains and Tulugou, respectively^54,55^, and severe drought in the 1920s–1930s was also obvious in Xinglong Mountain and Helan Mountain^27,32^, which further demonstrated the reliability of our reconstruction.

### Links with large-scale ocean-atmospheric circulations

It is widely accepted that ENSO plays a key role in regional moisture^28,56–58^, such as in the United States^59^, Southeast Asia^60^ and China^61,62^. Significant spectral peaks were identified at 2–3 years in our neighboring moisture-related tree-ring reconstructions, which were also attributed to the influence of ENSO on hydroclimate change^27,28,34^.

**Figure 8:**
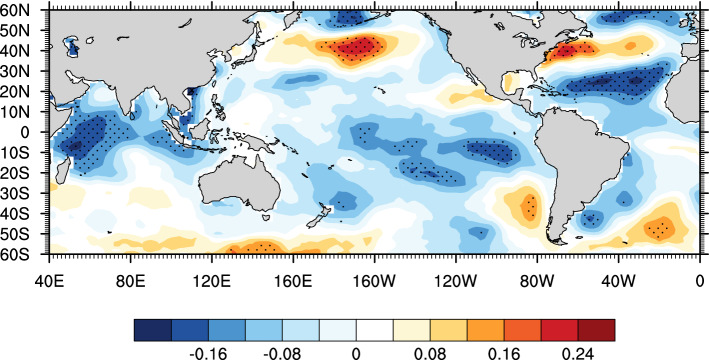
Spatial correlations of reconstructed series with concurrent SSTs for the period of 1870–2018. Dots indicate the correlation coefficients over a 90% confidence level. Figure is created using NCL version 6.6.2.

The results of spectral power analysis showed that significant spectral peaks were found at 2.1–3.8 years. Additionally, severe dry years in 1966, 1995, 1997 and 1998 corresponded to El Niño events^63^. Therefore, we confirmed the influence of ENSO on moisture change at our study site. A possible process is that ENSO influences the western North Pacific heating and South Asian heating during summer, which not only leads to changes in the atmospheric pressure pattern over the northwestern Pacific but also induces a meridional wave pattern from the tropical western North Pacific to mid-latitude East Asia and a zonal wave pattern over mid-latitude Asia^64,65^. As a result, during the El Niño phases, there is less precipitation and therefore moisture in north central China, and the opposite is found during the La Niña phases^66^. The spatial correlation results (Fig. 8) showed significantly negative correlations of the reconstructed scPDSI with the concurrent SST in the equatorial eastern Pacific Ocean during 1870–2018, which indicates that moisture variability declines during the El Niño phases, consistent with the previous findings mentioned above. In addition, significant spectral peaks at 24.9–46.5 years and significantly correlations patterns of GS with the SST in the Atlantic Ocean and the northern Pacific Ocean indicate the possible links between regional moisture change and the PDO and NAO at lower frequencies.

**Figure 9:**
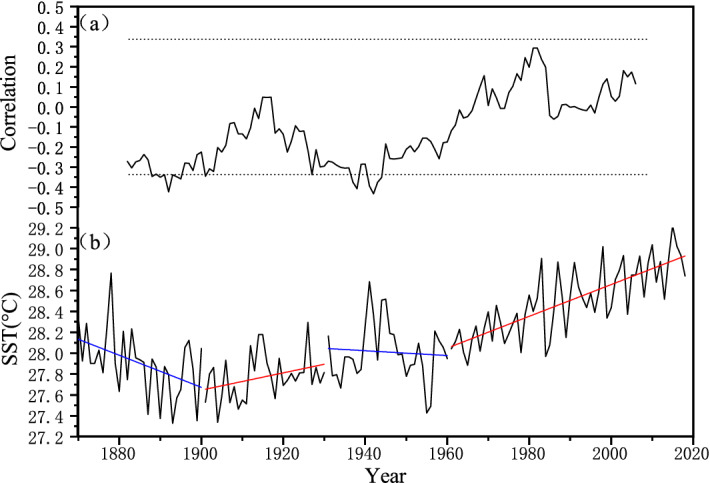
(**a**) Running 25-year correlations between reconstructed scPDSI and the ENSO index calculated using SST anomalies over NINO3.4. (**b**) Time series of mean SST in the tropical Indian Ocean (10° S–10° N and 50°–94° E) with linear trends for different periods. Dotted lines denote the 90% confidence level.

**Figure 10:**
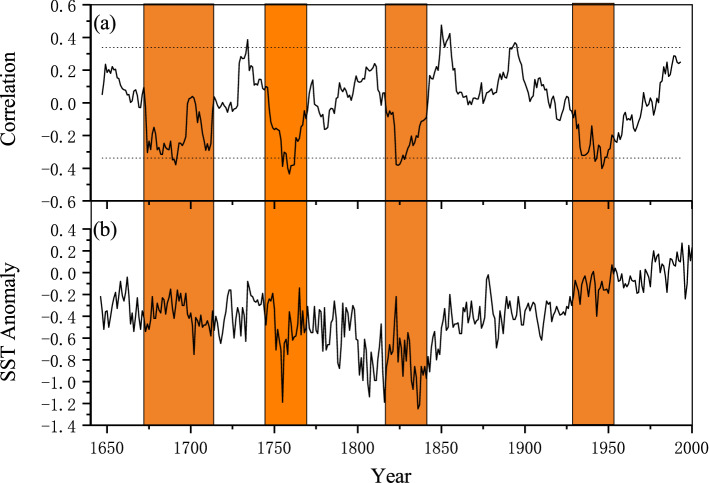
(**a**) Running 25-year correlations between reconstructed scPDSI and the ENSO index for 1635–2005. (**b**) Time series of mean SST reconstruction in the tropical Indian Ocean (15° S–20° N, 40°–100° E) for 1646–2001. Orange rectangles denote significant anticorrelation periods and the corresponding periods of decreasing SST in the tropical Indian Ocean. Dotted lines denote the 90% confidence level.

However, an increasing number of studies noted that the influence of ENSO on regional moisture change is time-dependent^67^. For example, Krishna et al.^68^ revealed that the relationship between the ENSO and Indian summer monsoon rainfall was inconsistent during the 1850s–1980s period. Similar findings were also observed in the East Asian summer monsoon region in recent decades^69^. To further understand the effect of ENSO on the moisture change in north central China, we computed 25-year running correlation coefficients of our reconstruction with a concurrent ENSO index. As shown in Fig. 9, the correlation coefficients fluctuated from 1870 to 2018. Therein, significant anticorrelations were found in the periods of 1870–1900 and 1931–1960, while nonsignificant correlations were found in 1901–1930 and 1961–2014. These results confirmed the notion of the unstable relationship between ENSO and moisture change in north central China. As previous studies indicated, the EASM has a strong biennial signal in its correlations with the tropical SST, and interannual variation in the summer atmospheric circulation is significantly different between the high- and low-correlation periods^69^. Therefore, it is reasonable to divide the whole period into several subperiods for further study. According to the correlation pattern shown in Fig. 9, the whole period was then divided into four subperiods: 1870–1900, 1901–1930, 1931–1960, and 1961–2014. A previous study found that the variation in SST in the tropical Indian Ocean might be responsible for the unstable ENSO-hydroclimate linkage in Southeast Asia at sub-centennial time scales^58^. Therefore, we calculated the mean SST in the tropical Indian Ocean (10° S–10° N and 50°–94° E). The results indicated that significant anticorrelations of the reconstructed scPDSI with the concurrent ENSO index occurred under the background of decreasing SST in the tropical Indian Ocean (Fig. 9b). Additionally, these relationships remained at longer timescales (Fig. 10). These findings demonstrated the modulation of different phases of SST in the tropical Indian Ocean on the ENSO-hydroclimate linkage in north central China.

**Figure 11:**
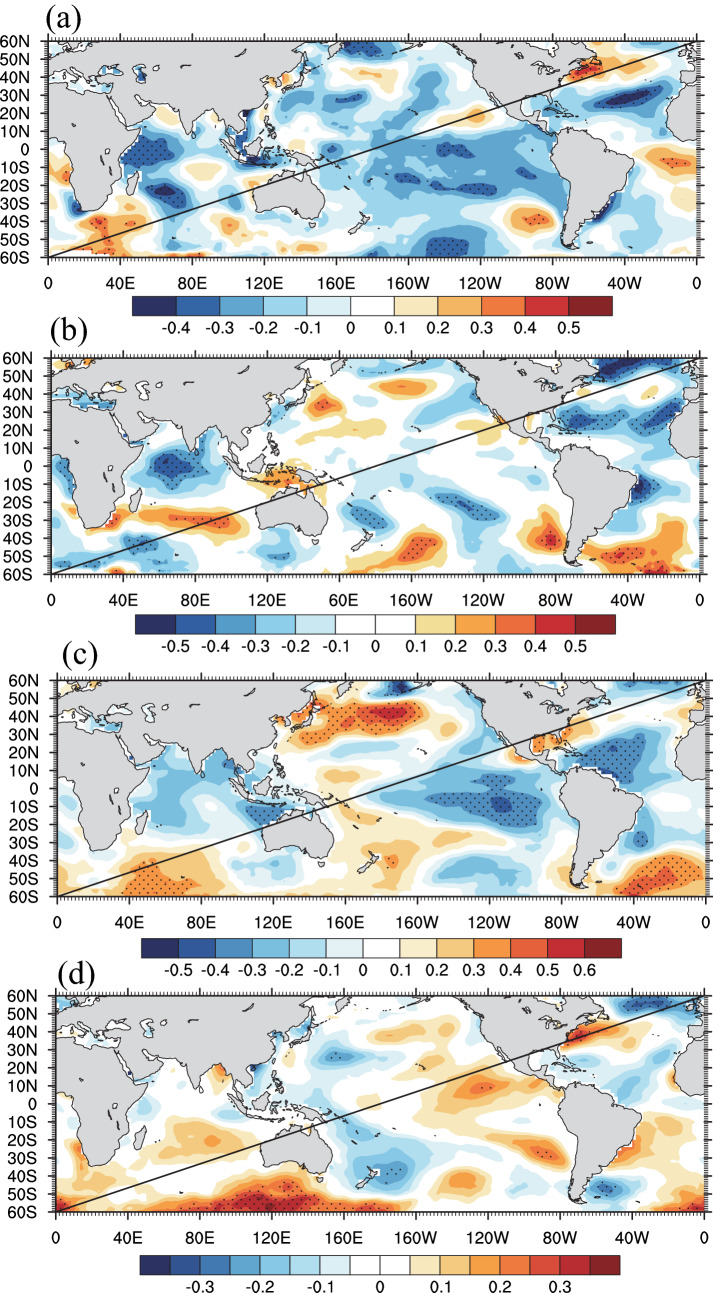
Spatial correlations between regional moisture and SSTs for the periods of (**a**) 1870–1900, (**b**) 1901–1930, (**c**) 1931–1960, and (**d**) 1961–2014. Dots indicate the correlation coefficients over the 90% confidence level. Figures are created using NCL version 6.6.2.

Thus, we surveyed the spatial relationship patterns of our reconstruction during these four subperiods with the concurrent SST (Fig. 11). Corresponding to Fig. 9, regional moisture was significantly positively correlated with SST in the eastern tropical Pacific Ocean during the periods of 1870–1900 and 1931–1960, which indicated that El Niño (La Niña) phases would result in dry (wet) conditions in north central China (Fig. 11a,c). In addition, during the period of 1901–1930 (Fig. 11c), the link of regional moisture with SST in the eastern tropical Pacific Ocean disappeared, while link of regional moisture with SST in the Indian Ocean remained the same as that during the periods of 1870–1900 and 1931–1960. However, during the period of 1961–2018 (Fig. 11d), the links of regional moisture with SST in the Indian Ocean and Pacific Ocean faded out. Previous studies found that SST anomalies in the Indian Ocean would induce Walker circulation anomalies and therefore affect the development of ENSO^70–72^. This “atmosphere bridge” connecting the Indian Ocean and Pacific Ocean might be the reason for this fading link of regional moisture and ENSO under the background of obvious warming in the Indian Ocean in recent decades.

Implications and limitations of the study. In this study, we developed a May–July moisture reconstruction with tree rings, spanning 1635–2018 in north central China. Our reconstruction was representative of large-scale May–July moisture change in north central China and revealed 4 severe dry epochs and 5 pronounced wet epochs during the past four centuries. The results enable us to better understand regional climate change under the background of global climate change. Furthermore, this study showed different forcing mechanisms for regional moisture variability, and particularly demonstrated a time-dependent relationship of regional moisture with ENSO, which was modulated by different phases of SST in the tropical Indian Ocean. These findings painted a history of ENSO-hydroclimate linkage and will decrease the difficulty of regional climate prediction using ENSO as a predictor in north central China. However, uncertainties exist and might affect our results. First, we employed total ring width rather than intra-ring sectors (earlywood width and latewood width) for reconstructing long-term moisture variations^73^. In some cases, intra-ring sectors provided stronger hydroclimatic signals than total ring width^74^. Additionally, we chose precipitation, temperature and scPDSI as major climatic factors, and of course, other climatic factors may also have had an impact on tree growth, such as total cloud cover and maximum temperature and so on^75,76^. Therefore, in future studies, more tree-ring parameters and climatic factors should be applied. Second, the results demonstrated the time-dependent relationship of regional moisture variation with ENSO, which was modulated by different phases of SST in the tropical Indian Ocean. However, this dynamic process requires future relevant investigations. Therefore, climate modeling experiments are required to understand the mechanisms behind the climate evolution of this period.

## Methods

### Climate condition in north central China

**Figure 12:**
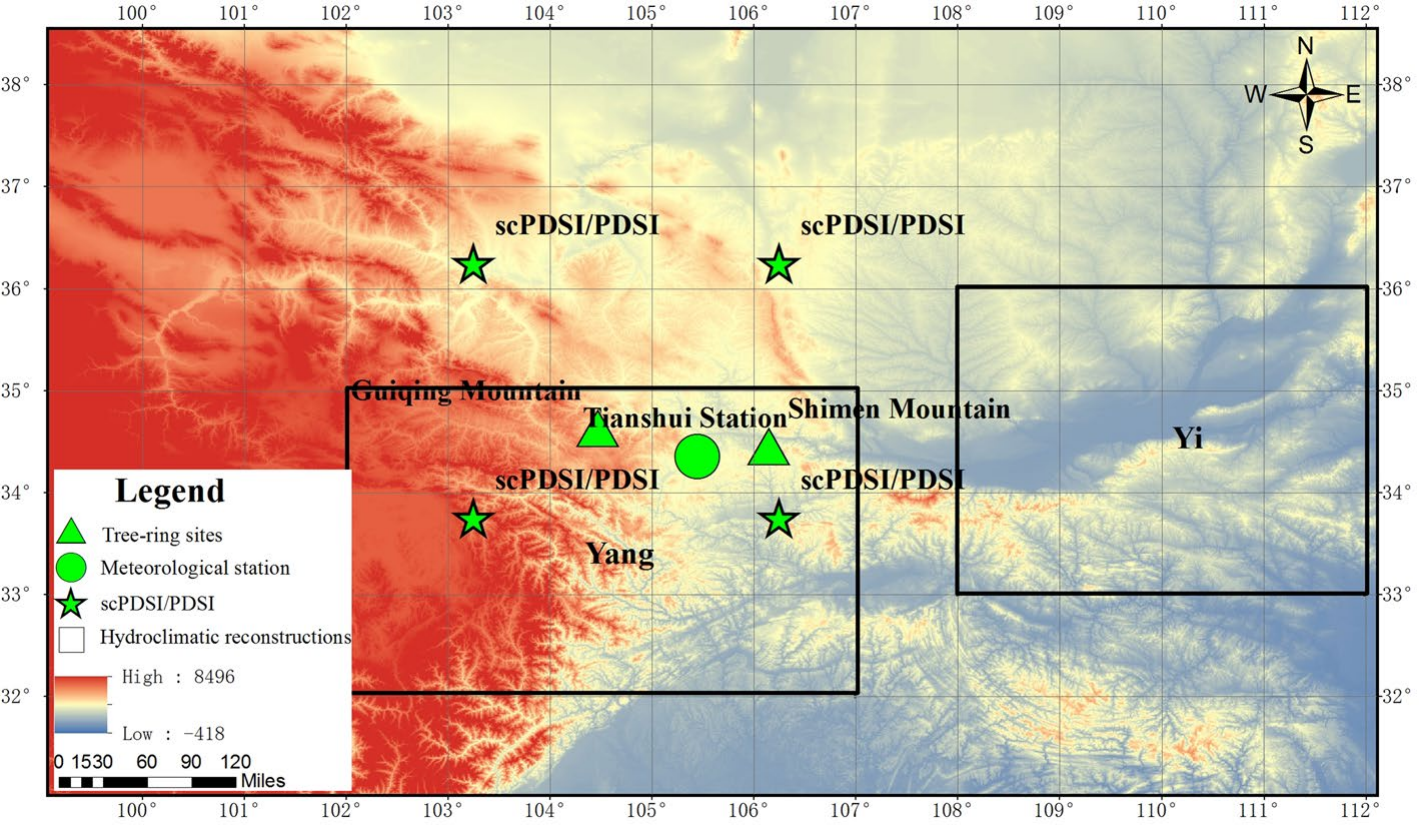
Locations of the sampling site, meteorological stations and comparison sites. Map is created using ArcGIS10.2 software for the ArcGIS Free Trial (https://lzuedu.maps.arcgis.com/).

Tree-ring samples were sampled in GQM and SMM, and the sample site elevations ranged from 2410 to 2540 m and from 2020 to 2180 m, respectively (Fig. 12). These two sites are close to each other and situated in the transition zone between the arid and humid regions. As Tianshui station recorded, the nearest meteorological station among these two sites, the annual mean maximum temperature, temperature, minimum temperature and total precipitation were 17.2 °C, 11.1 °C, 6.6 °C and 515.1 mm, respectively, for 1951–2019, with peak warm and wet conditions in July.

### Climate data

The annual mean temperature and total precipitation were calculated from monthly mean data for the period of 1951–2019 derived from Tianshui station, downloaded from the China Meteorological Administration (http://data.cma.cn). The scPDSI^53^, with a 2.5° × 2.5° resolution, was used as a criterion for drought. The regional scPDSI was calculated by averaging 4 gridded data over the area between 33.75° N–36.25° N and 103.75°–106.25° E. In addition, the regional June–August PDSI, which came from MADA, and was calculated by averaging the same area was used for comparing. These data were downloaded from the National Oceanic and Atmosphere Administration (https://www.noaa.gov/). The SST dataset from the National Oceanic and Atmospheric Administration (NOAA) Extended Reconstructed SST V5 was downloaded from https://www.noaa.gov/. Based on these data, we calculated the monthly ENSO index over the Niño3.4 region (120°–170° W, 5° S–5° N). Paleoclimatology data, including ENSO index reconstruction, hydroclimatic reconstructions based on tree-ring data and SST reconstruction over the tropical Indian Ocean, were downloaded from the U.S. National Centers for Environmental Information (https://www.ncdc.noaa.gov/data-access/paleoclimatology-data).

### Tree-ring material and chronology development

We sampled one or two cores from living trees of Chinese pine at breast height (1.3 m above ground) with 10-mm increments in GQM in 2018 and with 5-mm increments in SMM in 2019. In total, 46 cores from 26 trees in GQM and 38 cores from 22 trees in SMS were collected. In accordance with the standard procedure for analyzing tree rings, pretreatments were conducted. First, all cores were properly mounted, dried and sanded in the laboratory^77^. Thereafter, preliminary dating was conducted using an optical microscope. Then, ring widths were measured at 0.001 mm precision using a Velmex ring-width measuring system. After that, the program COFECHA was used for quality control of crossdating^78^. The raw ring widths were then detrended using a negative exponential curve and cubic smoothing spline with a 50% frequency cut-off in the program ARSTAN^79^. Finally, a STD, a residual chronology (RES) and an autoregression chronology (ARS) were obtained. We chose STD for further study due to its good quality of maintaining low-frequency signals.

### Statistical analyses

Correlation analysis was performed to determine the relationship between ring-width index and climatic factors. A linear regression model^41^, which was later verified using the split calibration-verification test method^40^, was used for the reconstruction. Spatial correlation analyses were conducted to detect whether our reconstruction showed spatiotemporal representativeness. Additionally, visual comparison was performed to examine the reliability of our reconstruction backwards in time. Furthermore, MTM analysis was performed to probe the cycle of our chronology using Mike Mann’s spectral analysis signal reconstruction program downloaded from Lamont-Doherty Earth Observatory (https://www.ldeo.columbia.edu/tree-ring-laboratory/resources/software)^42^. To detect the influence of SST on regional moisture, running correlation analyses and spatial correlation analyses were performed. Spatial correlation analyses were performed using NCAR Command Language (NCL; https://www.ncl.ucar.edu/). Detailed in Fig. 7 is created using Origin 2021 for trial version. Detailed in Fig. 12 is created using ArcGIS10.2 software for the ArcGIS Free Trial (https://lzuedu.maps.arcgis.com/).
